# Prone vs supine percutaneous nephrolithotomy: does position affect renal pelvic pressures?

**DOI:** 10.1007/s00240-024-01555-6

**Published:** 2024-04-17

**Authors:** Ala’a Farkouh, Kyu Park, Matthew I. Buell, Nicole Mack, Cliff De Guzman, Toby Clark, Elizabeth A. Baldwin, Kanha Shete, Rose Leu, Akin S. Amasyali, Evan Seibly, Kai Wen Cheng, Sikai Song, Zhamshid Okhunov, D. Duane Baldwin

**Affiliations:** Department of Urology, Loma Linda University Health, Room A560, 11234 Anderson Street, Loma Linda, CA 92354 USA

**Keywords:** Percutaneous nephrolithotomy, Patient positioning, Pressure, Kidney calculi, Prone position, Supine position

## Abstract

The purpose of this study was to measure and compare renal pelvic pressure (RPP) between prone and supine percutaneous nephrolithotomy (PCNL) in a benchtop model. Six identical silicone kidney models were placed into anatomically correct prone or supine torsos constructed from patient CT scans in the corresponding positions. A 30-Fr renal access sheath was placed in either the upper, middle, or lower pole calyx for both prone and supine positions. Two 9-mm BegoStones were placed in the respective calyx and RPPs were measured at baseline, irrigating with a rigid nephroscope, and irrigating with a flexible nephroscope. Five trials were conducted for each access in both prone and supine positions. The average baseline RPP in the prone position was significantly higher than the supine position (9.1 vs 2.7 mmHg; *p* < 0.001). Similarly, the average RPP in prone was significantly higher than supine when using both the rigid and flexible nephroscopes. When comparing RPPs for upper, middle, and lower pole access sites, there was no significant difference in pressures in either prone or supine positions (*p* > 0.05 for all). Overall, when combining all pressures at baseline and with irrigation, with all access sites and types of scopes, the mean RPP was significantly higher in the prone position compared to the supine position (14.0 vs 3.2 mmHg; *p* < 0.001). RPPs were significantly higher in the prone position compared to the supine position in all conditions tested. These differences in RPPs between prone and supine PCNL could in part explain the different clinical outcomes, including postoperative fever and stone-free rates.

## Introduction

In recent years, there has been a growing interest in renal pelvic pressure (RPP) during endourologic procedures, including ureteroscopy and percutaneous nephrolithotomy (PCNL). Elevated pressures may lead to pyelovenous backflow, systemic fluid absorption, forniceal rupture, and kidney damage [1–3]. Conversely, low RPPs are hypothesized to cause increased blood loss due to less tamponade on bleeding venules, and collapse of the pelvicalyceal system, which can subsequently limit visibility [4].

Several factors have been reported to affect RPP. In ureteroscopy, these include the presence, position, and size of ureteral access sheaths, the type of irrigation used, and the use of working channel accessories [2]. For PCNL, the use of multiple tracts, the addition of suction, and the type of nephroscope can all affect RPPs and subsequent patient outcomes [4]. One factor that may also affect RPP during PCNL is patient position.

Traditionally, PCNL has been performed in the prone position, however, supine PCNL is currently gaining popularity. Although it has been theorized that supine PCNL results in lower RPPs, to date, there has been no objective comparison to prone PCNL. The purpose of this study was to measure and compare RPPs during prone and supine PCNL in a benchtop model.

## Materials and methods

**Fig. 1:**
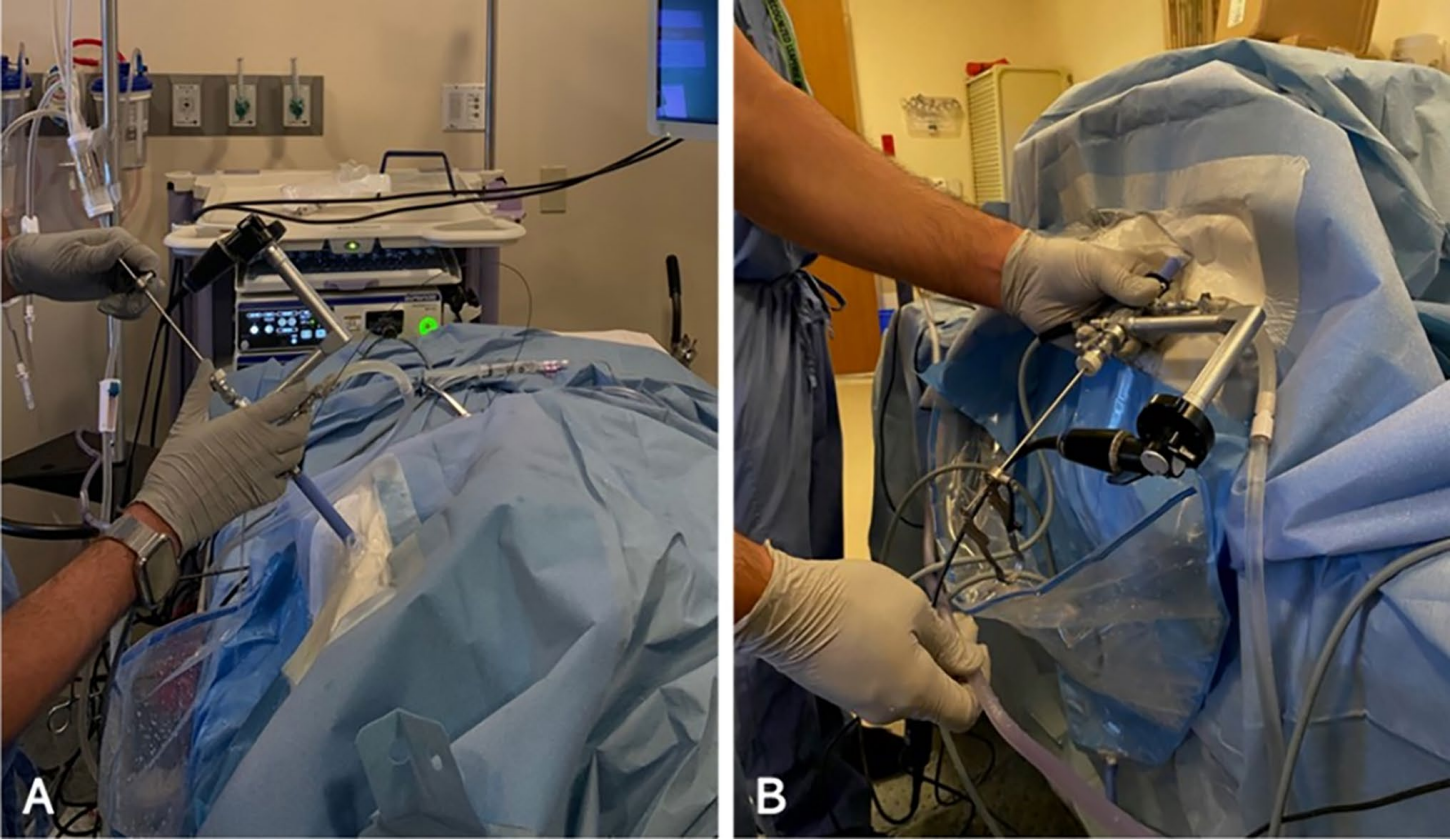
Experimental setup showing simulated PCNL in the **A** prone and **B** supine positions

This was a benchtop study designed to measure RPP during simulated PCNL using kidney models and phantom torsos in prone and supine positions (Fig. 1). No human subjects were involved in this study and thus it was exempt from Institutional Review Board approval.

Three-dimensional (3D) kidney models were constructed from an actual patient CT scan of the right kidney. Initially, the CT image was downloaded as a Digital Imaging and Communications in Medicine (DICOM) format, uploaded onto 3D Slicer software [5], and converted to Nearly Raw Raster Data (NRRD) format. Next, Embodi3D (Embodi3D, Bellevue, WA) and Meshmixer (Autodesk, San Rafael, CA) programs were employed to remove the surrounding tissues and bones, allowing the kidney and collecting system to be isolated. Autodesk Inventor (Autodesk, San Rafael, CA) was then used to create a negative mold of a coronally bisected right kidney with a complete collecting system. Subsequently, Cura software was used to create the g-code from which the mold was printed using an Ultimaker 3 3D printer (Ultimaker, Utrecht, Netherlands).

The mold was then filled with Dragon Skin™ 20 silicone rubber (Smooth-On, Inc., Macungie, PA) and 12 coronally bisected half silicone kidneys were created. A 30-cm long polyvinyl chloride tube with 4.3-mm inner diameter was placed at the position of the hilum. Two silicone kidney halves along with one tube were sealed together using additional Dragon Skin™ 20 silicone rubber to create a water-tight kidney and ureter model. A total of six identical kidney and ureter models were created in this fashion.

To construct the torso models, a Laerdal patient manikin torso (Laerdal Medical, Stavanger, Norway) was encased with plaster rolls dipped in warm water and left to dry for 24 h until completely hardened. Two torso molds were created in this manner, one to simulate prone position and one to simulate supine. For supine positioning, two rolled towels were placed under the torso during the experiment to simulate actual positioning and angles during PCNL.

Using two different CT scans of the same patient in both prone and supine positions, the following measurements were taken in order to position the kidney within the model: right apex to posterior wall, right apex to lateral wall, right bottom to posterior wall, and right bottom to lateral wall. These measurements were then translated onto wooden dowels and were cut accordingly to position the kidney models. Once the silicone kidneys were positioned appropriately in both the prone and supine models, Big Gap Filler (Great Stuff, Austin, TX) was used to fill in the remaining plaster cavity and left to dry in between layers.

**Fig. 2:**
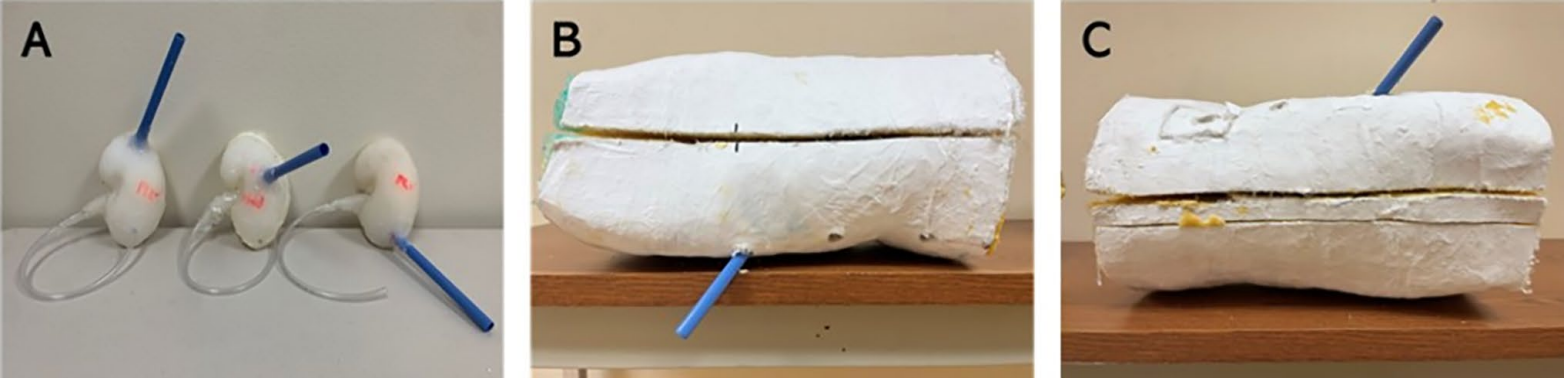
**A** Three silicone kidney and ureter models for use in prone PCNL with renal access sheaths fixed in the upper, middle, and lower poles. **B** External view of supine torso model with upper pole access. **C** External view of prone torso model with upper pole access

After confirming anatomic positioning, one 30-Fr Amplatz renal sheath (Boston Scientific, Marlborough, MA) was placed in each silicone kidney through the torso model in a specific site. Three access sites were achieved in each of the prone and supine positions, including upper pole, middle pole, and lower pole. All renal accesses were directed by an endourologist to simulate the position of access for the corresponding procedure. Dragon Skin™ 20 silicone rubber was used to fix the Amplatz sheath to the silicone kidney model. Figure 2 shows the three kidney models used for prone PCNL and the two torso models used in the study.

To measure RPP, a 7.95-Fr URF-P6R flexible ureteroscope (Olympus, Tokyo, Japan) was positioned inside the ureter tubing with its tip just inside the renal hilum. The ureteroscope working channel was connected to an arterial line transducer and pressure monitor that measured pressure in mmHg, as in a previously described manner [6]. At each position and access site, RPP was measured under three conditions: baseline, during irrigation through a 26-Fr rigid nephroscope, and during irrigation through a 16-Fr flexible nephroscope.

Five trials were conducted to measure RPP for each condition in each unique position and access site, giving a total of 90 trials. For each trial, two 9-mm BegoStone spheres (Bego USA, Lincoln, RI) were placed in the renal pelvis to simulate nephrolithiasis. For all trials, saline irrigation was fixed at a height of 90-cm from the bottom of the bag to the position of the kidney.

All statistical analyses were performed using R Statistical Software v4.1.2 (R Core Team 2021). The Wilcoxon Rank-Sum was used to compare prone and supine RPPs, while the Kruskal–Wallis test followed by Dunn’s test was used to compare pressures between different access sites. For all comparisons, a *p*-value of < 0.05 was considered significant.

## Results

At baseline, when combining data from all three access sites, the average RPP was 3.4 times higher in the prone position compared to the supine position (9.1 vs 2.7 mmHg; *p* < 0.001). When comparing baseline pressures in the prone position based on access site, utilizing the lower pole resulted in lower RPP (5.2 mmHg) compared to the upper (10.2 mmHg) and middle (12.0 mmHg) poles, but this was not statistically significant (*p* > 0.05). There was no difference in RPPs between the upper, middle, and lower poles in the supine position at baseline (2.4 vs 3.4 vs 2.2 mmHg respectively; *p* > 0.05).

When using the rigid nephroscope with irrigation, the average RPP from all three access sites was also 3.6 times higher in the prone position compared to the supine position (19.1 vs 5.3 mmHg; *p* < 0.001). Similarly, utilizing the flexible nephroscope with irrigation resulted in significantly higher RPPs in the prone position compared to the supine position (13.6 vs 1.6 mmHg; *p* < 0.001).

When comparing working pressures based on renal access sites, there was no significant difference in pressures in either the prone position (upper = 15.9 mmHg, middle = 20.0 mmHg, lower = 13.1 mmHg; *p* > 0.05) or the supine position (upper = 3.2 mmHg, middle = 4.2 mmHg, lower = 2.7 mmHg; *p* > 0.05). In all working conditions, utilizing a rigid nephroscope resulted in significantly higher pressures compared to a flexible nephroscope in both prone (19.1 vs 13.6 mmHg; *p* = 0.002) and supine positions (5.3 vs 1.6 mmHg; *p* < 0.001).

**Fig. 3:**
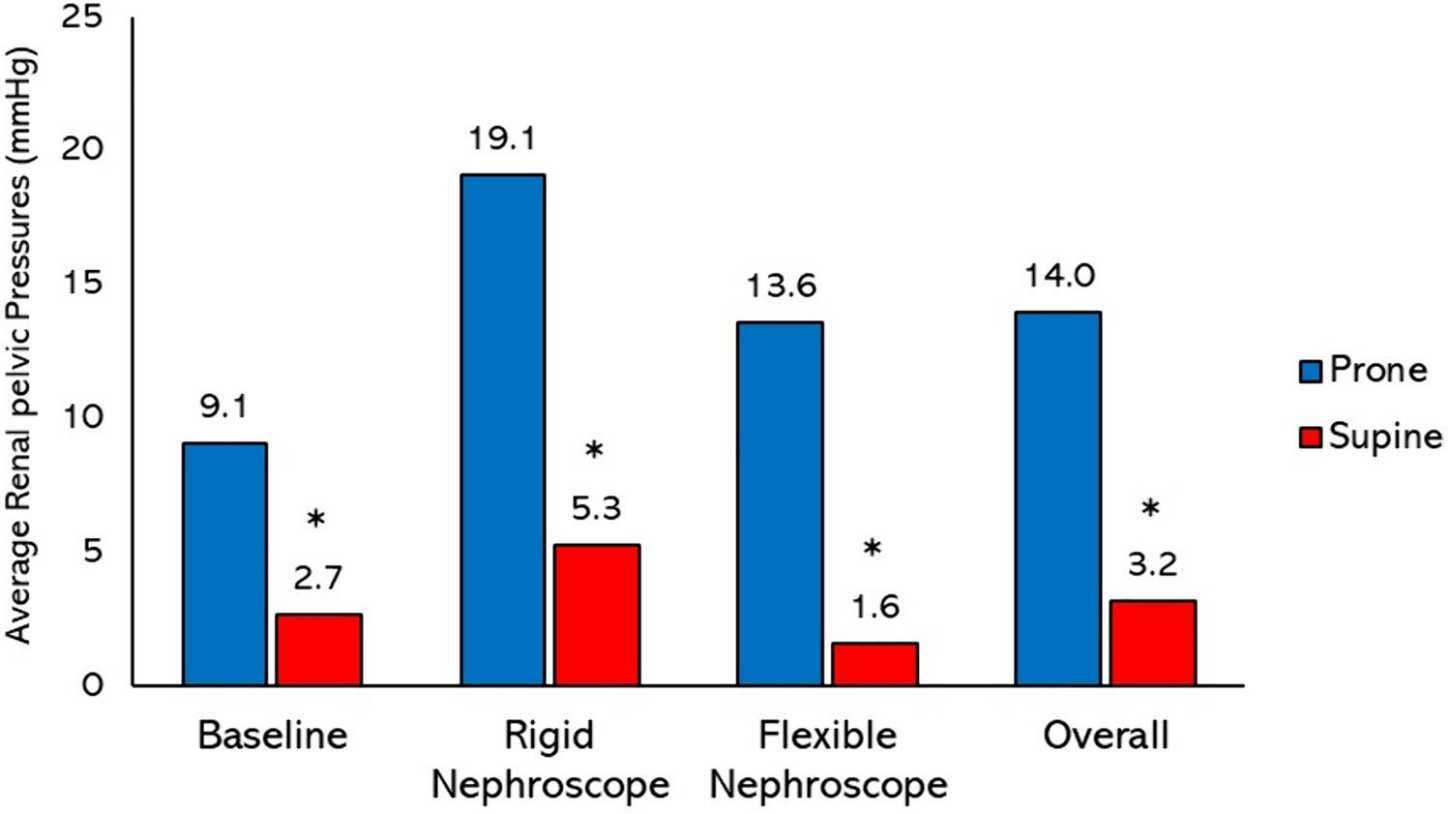
Variation in renal pelvic pressures between prone and supine PCNL combining all three renal access locations. Supine PCNL consistently resulted in lower renal pelvic pressures (* indicates significant difference with *p* < 0.05)

Overall, when combining all RPPs at baseline and with irrigation, with all access sites and types of scopes, the mean pressure was significantly higher in the prone position compared to the supine position (14.0 vs 3.2 mmHg; *p* < 0.001). This translates into an average 4.4 times higher RPP in prone PCNL compared to supine PCNL. Figure 3 summarizes the RPPs in prone and supine PCNL using data from all access sites under the various conditions tested.

## Discussion

Recently, the importance of RPP during PCNL has been implicated as a potential cause of complications including fever, pain, and kidney injury [6–8]. Although the study of RPP is in its infancy, the upper threshold for RPP is often set at 30 mmHg (sometimes reported as 40 cmH_2_O), as pyelovenous backflow is estimated to occur when pressures exceed this level [1, 6, 9]. In our previous clinical study, we measured RPPs in patients undergoing PCNL, and found that postoperative pain scores and hospital lengths of stay were significantly higher if RPPs exceeded 30 mmHg [6]. Similarly, Wu et al. and Zhong et al. reported that an accumulated time of 40–60 s with RPPs > 30 mmHg led to a significantly higher incidence of postoperative fever after mini-PCNL [7, 8].

Although there has been research on elevated RPP, the significance and implications of extremely low RPP are not well elucidated. Having extremely low RPPs can lead to collapse of the pelvicalyceal system, which may limit the surgeon’s visibility and prevent adequate stone clearance [4]. In addition, a collapsed system confers a higher risk of urothelial mucosal injury during ultrasonic or laser lithotripsy and during suction. Any ensuing bleeding would also be more difficult to control due to less tamponade on bleeding capillaries and venules. Therefore, in PCNL, the optimal RPP would be high enough to allow safe and effective surgery, but not too high to cause complications resulting from pyelovenous or pyelolymphatic backflow.

In our current study, we investigated patient position during PCNL to determine the effect upon RPP. We demonstrated that under controlled settings, RPPs are indeed higher during prone PCNL compared to supine PCNL, but in all conditions tested, pressures never exceeded 30 mmHg. This study also found that in either position, the site of renal access (upper, middle, or lower) does not lead to significant alterations in RPP. Additional findings of our study include a 30–70% lower RPP when utilizing a flexible nephroscope compared to a rigid nephroscope. These lower pressures with flexible nephroscopy are in line with prior studies measuring RPPs during prone PCNL in both porcine models and patients during PCNL [4, 6].

Supine percutaneous renal access was first performed and described in 1987 by Valdivia Uria, who subsequently published on a series of 520 patients who underwent supine PCNL [10]. Over the years, many urologists have published their experience with supine PCNL and compared surgical and patient outcomes to prone PCNL. Notably, a prospective multicenter global study compared outcomes between prone and supine PCNL in 5775 patients and reported significantly higher stone-free rates with prone PCNL (77.0% vs 70.2%; p < 0.0001). However, they also found that prone PCNL had significantly higher fever rates (11.1% vs 7.6%; p < 0.001) [11]. Similarly, Kasap et al. reported that infective complications occurred at a significantly higher rate after prone PCNL compared to supine PCNL (18% vs 7.5%; p = 0.034), and prone position was found to be an independent risk factor for postoperative infections (OR = 4.5; p = 0.02) [12].

These infections are hypothesized to be due to higher RPPs in prone PCNL causing more pyelovenous backflow and leading to increased bacterial translocation into the bloodstream [12]. Although pressures never exceeded 30 mmHg in our benchtop study, we have indeed confirmed that RPP is higher in prone PCNL when compared to supine and this may partially explain the higher rates of infections with prone PCNL. As previously mentioned, our understanding of RPP is still in its infancy and other conflicting studies have not demonstrated increased fever with prone PCNL. A prospective randomized study by Al-Dessoukey et al. compared 102 prone PCNL patients to 10 l supine PCNL patients and reported no significant difference in fever rates (5.9% vs 5%; *p* = 0.77) [13]. Similarly, Wang et al. found no difference in postoperative fever between prone and supine PCNL [14].

With regards to stone-free rates, Al-Dessoukey et al. did not find a difference between prone and supine PCNL (87.3% vs 88.1%; *p* = 0.85) [13], however, Wang et al. reported that stone-free rates were significantly lower with supine PCNL compared to prone PCNL (73.3% vs 88.7%; *p* = 0.03), with 10% of patients in the supine group requiring a second look [14]. Conflicting results have also been reported by several meta-analyses. In one analysis comparing prone and supine PCNL, 13 studies were included, and stone-free rates were significantly lower in supine PCNL (OR = 0.74; *p* < 0.001) [15]. However, two other meta-analyses showed no difference in stone clearance between prone and supine PCNL [16, 17]. One recently published study specifically compared mini-PCNL in the prone and supine positions and did not find any differences in either stone-free rates or complication rates, but found that both 6-h and 24-h pain scores were significantly lower in supine mini-PCNL compared to prone (*p* < 0.001) [18].

Performing PCNL in the supine position has several reported advantages [19–21]. Patient positioning is less complicated and less time-consuming as there is no need to turn patients over. This is particularly important in patients with contractures or mobility issues and in morbidly obese patients. Having patients in the supine position also facilitates airway management and maintenance of cardiovascular function. In supine PCNL, the renal access tract is more horizontal in position allowing for spontaneous stone fragment evacuation and more fluid drainage, explaining the lower RPPs observed in our study. This also explains the faster operative times reported by some studies [15, 16]. In addition, less fragments will migrate down the ureter in supine PCNL due to the lower position of the kidney in relation to the ureter, also explaining the shorter operative times.

Additional advantages that have been described for supine PCNL over prone PCNL include the feasibility of endoscopic combined intrarenal surgery (ECIRS), as well as having the surgeon’s hands outside of the primary radiation beam due to the location of the access site and tract [21]. However, these can be successfully overcome in prone PCNL. In one study, prone split-leg position with ureteroscopy allowed for successful renal access using an ECIRS approach in a series of patients [22]. Laser-guided access can significantly reduce fluoroscopy time, allowing needle insertion in the prone position without the need for continuous fluoroscopy [23]. Use of needle holders can also successfully guide needle insertion during prone PCNL with significantly reduced radiation exposure to the surgeon’s hand and to the patient [24].

Conversely, supine PCNL has several drawbacks over prone PCNL. Having patients in the supine position leads to a narrower surgical field and window of access into the kidney. Upper pole access is particularly challenging in supine PCNL, due to its more medial and posterior location, and there is a higher risk of spleen and liver injury [19, 25]. In another prospective study reporting specifically on 1311 patients with staghorn calculi, there was significantly higher upper pole access in the prone group (12.6% vs 3.6%; *p* < 0.001), significantly longer operative times with supine (123.1 vs 103.2 min; *p* < 0.001), and higher stone-free rates in prone PCNL (59.2% vs 48.4%; *p* < 0.001) [26]. The challenges with limited upper pole access, together with the low pressures and system collapse seen in supine PCNL, may explain these longer durations and lower clearance rates in patients with complex staghorn stones. Additional implications include longer tract lengths in supine PCNL that may limit maneuverability, which becomes even more difficult in obese patients due to more adipose tissue and longer tracts [27, 28].

Ultimately, the decision to perform PCNL in the prone or supine positions depends on both surgeon and patient factors. These include surgeon preference, training, and experience as well as patient habitus, stone burden and location, and the presence of certain congenital anomalies that may preclude the ability to perform PCNL in a specific position. Despite the increasing popularity of supine PCNL, prone PCNL still remains the most common position, with 47.5% of urologists utilizing prone position exclusively, 16.3% using supine exclusively, and 36% using both as reported by the recent Endourological Society global census [29]. Regardless of their chosen position, it is important that surgeons remain cognizant of the advantages and drawbacks of either position, and how the differences in RPP may affect outcomes.

Limitations of this study include its benchtop design and reliance on kidney models which do not accurately reflect the distensibility of the human kidney and pelvicalyceal system. Outcomes that are influenced by the differences in pressure could not be assessed in our study, including impaired visibility, more bleeding, stone clearance, and infectious complications. In addition, patient factors that could influence positioning or RPPs were not accounted for in this study, such as body habitus, stone burden, and anatomic variations of the kidney. Finally, technical differences and the use of various instruments that can impact RPP were not investigated in this study. Despite these limitations, our study was able to accurately document the magnitude of RPPs in both prone and supine positions for PCNL, utilizing different access sites and different types of endoscopes in a controlled setting with fixed measurements, irrigation settings, and testing environment.

## Conclusions

Prone PCNL had significantly higher RPPs at baseline and during irrigation with both rigid and flexible nephroscopes compared to supine PCNL. These higher pressures may partially explain the increased incidence of fever and infections seen after prone PCNL. Conversely, the lower pressures in supine PCNL may partially explain the reported lower stone-free rates due to system collapse and impaired visibility. Knowledge of the effect of patient positioning upon RPP could be one factor that helps surgeons guide the optimal position for PCNL in an individual patient.
